# Cinemeducation in medicine: a mixed methods study on students’ motivations and benefits

**DOI:** 10.1186/s12909-022-03240-x

**Published:** 2022-03-12

**Authors:** Mike Rueb, Matthias Siebeck, Eva A. Rehfuess, Lisa M. Pfadenhauer

**Affiliations:** 1 Pettenkofer School of Public Health, Elisabeth-Winterhalter-Weg 6, 81377 Munich, Germany; 2 Institute for Medical Information Processing, Biometry and Epidemiology, LMU Munich, Elisabeth-Winterhalter-Weg 6, 81377 Munich, Germany; 3 Department of Psychiatry and Psychotherapy, University Hospital, LMU Munich, Nussbaumstrasse 7, 80336 Munich, Germany; 4 Institute of Medical Education, University Hospital, LMU Munich, Pettenkoferstrasse 8a, 80336 Munich, Germany

**Keywords:** Cinemeducation, Medical cinema, Medical humanities, Biopsychosocial model, Health professional education, Interprofessional education, Film and medicine, Movies and medicine

## Abstract

**Background:**

Cinemeducation courses are used to supplement more standard teaching formats at medical schools and tend to emphasise biopsychosocial aspects of health. The purpose of this paper is to explore why medical students attend the cinemeducation course M23 Cinema (M23C) at LMU Munich and whether a film screening with a subsequent expert and peer discussion benefits their studies and their future careers as medical doctors.

**Methods:**

An exploratory sequential mixed methods study design was used. Qualitative research, i.e. three focus groups, four expert interviews, one group interview and one narrative interview, was conducted to inform a subsequent quantitative survey. Qualitative data was analysed using qualitative content analysis and quantitative data was analysed descriptively. The findings were integrated using the “following a thread” protocol.

**Results:**

In total, 28 people were interviewed and 503 participants responded to the survey distributed at seven M23C screenings. Participants perceive the M23C as informal teaching where they learn about perspectives on certain health topics through the combination of film and discussion while spending time with peers. The reasons for and reported benefits of participation varied with educational background, participation frequency and gender. On average, participants gave 5.7 reasons for attending the M23C. The main reasons for participating were the film, the topic and the ability to discuss these afterwards as well as to spend an evening with peers. Attending the M23C was reported to support the students’ memory with regards to certain topics addressed in the M23C when the issues resurface at a later stage, such as during university courses, in the hospital, or in their private life.

**Conclusions:**

The M23C is characterised by its unique combination of film and discussion that encourages participants to reflect upon their opinions, perspectives and experiences. Participating in the M23C amplified the understanding of biopsychosocial aspects of health and illness in students. Thus, cinemeducative approaches such as the M23C may contribute to enabling health professionals to develop and apply humane, empathetic and relational skills.

**Supplementary Information:**

The online version contains supplementary material available at 10.1186/s12909-022-03240-x.

## Background

To this day, the curricula of medical faculties reflect the prevailing paradigm of health and disease in the Western world: the biomedical model. This model, however, has been scrutinised with regards to its inability to integrate the complexities of health and disease and neglecting the psychosocial aspects of medicine [1]. Engel argued for a more inclusive scientific model of medicine, defined as the biopsychosocial model [2, 3], which was subsequently embraced by the medical humanities [4]. This field promotes interdisciplinary teaching and research to achieve a deeper understanding of human health and illness with the ultimate goal of improving patient care [5].

This thinking of including behavioural and social factors in medical education clearly constitutes a shift in how health and disease are perceived. The probability that this perspective will also be applied by future doctors is greater if biopsychosocial determinants are already taught in medical school [6]. As for medical teaching in general, didactical approaches for teaching biopsychosocial perspectives on health vary widely [7]. One approach is to employ the medium of film [8–11], a didactical concept referred to as cinemeducation [12]. Cinemeducation courses in various forms are part of some medical schools’ curricula. Some use film clips, others show whole films in seminars and discuss them afterwards [13, 14]; most courses are obligatory or an elective, some courses are voluntary [10, 15–18]. Cinemeducation has been used to teach medical professionalism [14], medical ethics [18, 19], doctor-patient communication [13], emotions [9, 20], empathy [21, 22] or pharmacology [23] to medical students. Furthermore, films might be an opportunity for health professionals to gain a deeper understanding of the meaning of belonging to a care-providing, helping profession [24]. Research was also used to explore the nature of medical topics that could be taught through films [9, 13, 25–29].

### M23 cinema course description

At LMU Munich, the cinemeducation course M23 Cinema (M23C) – in German “Modul-23-Kino” or colloquially “M23-Kino” – is offered as a voluntary course at the Medical Faculty. M23 refers to module 23, the basic clinical year of the Medical Curriculum Munich (MeCuM), from which the M23C originally emerged. It is targeted at medical students in pre-clinical and clinical training as well as all other students of the health professions (e.g. psychology, nursing, social work, physiotherapy, dentistry, public health, medical technical assistants). It is also increasingly attended by non-medical students. M23C occurs on three to five evenings during the term and usually lasts 2.5 h. It takes place in a large lecture hall at the Medical Faculty of LMU Munich with up to 300 seats. In M23C, a film concerned with a specific health topic is shown, followed by an expert and peer discussion between one to four experts and/or patients and/or their relatives and the audience. Established in 2005, the M23C is currently being organised by a committee of medical students across all semesters who decide on the topics, films and experts for each event. The focus is on topics which – from the students’ point of view – are not sufficiently covered in the curriculum and which would benefit from being discussed from multiple perspectives (e.g. intersexuality, transgender, abortion, surrogacy, euthanasia, Pompe disease, fast food, stigma about psychiatry, amputation). There is no restriction on the types of film (e.g. feature films, documentaries or short films). For the discussion, experts and, if possible, those affected by the topic (e.g. patients, relatives) are invited. The event is moderated by a medical student from the organising committee with questions raised mainly by the audience.

What distinguishes M23C from other cinemeducation courses is (1) the structure of M23C with the division into film screening and audience discussion, (2) that it is a voluntary evening event and (3) that in M23C the learning objectives as well as the questions to the experts and stakeholders are not predefined. The learning objectives are decided upon by the learners themselves and can be addressed by raising questions in the discussion.

Despite the fact that the M23C was established more than 15 years ago and is widely attended by between ten and 300 students per event, it is still not clear what motivates students and others to participate in these events and how the M23C enables learning.

The first objective of this study was therefore to assess the reasons that students attend the M23C on a voluntary basis. In particular, we investigated whether these reasons vary between different groups of participants. The second objective was to assess how students benefit from attending the M23C. We explored whether and how these benefits played out in their ongoing education. Until the study was conducted, there was only speculation about the reasons for participating in M23C and what the benefits might be. By knowing the reasons for and benefits of attending the M23C, we wanted to find out to what extent psychosocial aspects matter in M23C. As for the current generation of medical students, working and learning with film can encourage interest, enthusiasm and creativity [17], we wanted to provide arguments why cinemeducation courses should play a broader role in medical curricula.

## Methods

### Setting

This study was conducted at the medical faculty of the LMU Munich in Germany. The qualitative component of the study took place from October 2016 until February 2017. The quantitative component took place in the summer term 2017 and winter term 2017/2018.

### Literature search

We conducted non-systematic literature searches in MEDLINE, PUBMED, PsycINFO, Psyndex and ERIC with the key words „cinema“, „medical cinema“, „film and medicine“ and „cinemeducation“, followed by additional searches of the reference lists of relevant articles. We used this literature to (i) inform the design of this study and, importantly, to (ii) design the guides for qualitative interviews and focus group discussions as well as the questionnaire.

### Mixed methods study

We first wanted to explore the topics within the complex M23C course before deciding what variables needed to be measured. Therefore, we used a mixed methods approach [30, 31] with an exploratory sequential design [32], where the qualitative component of the study preceded the quantitative component of the study and was used to inform quantitative data collection. The qualitative component entailed three focus group discussions (FGDs), four expert interviews, one group interview and one narrative interview. The quantitative component entailed a survey.

### Qualitative sampling and recruitment

For the qualitative component, we employed purposive sampling [33]. The main inclusion criterion for focus groups was that participants had taken part in at least two cinema evenings. Inclusion criteria for expert interviews were that participants had taken part in one M23C evening in the last 12 months and that we could interview an affected person and an expert from the same event. Participants were approached via e-mail, a faculty newsletter and the M23C social media page.

### Qualitative data collection

The data collection was undertaken by two researchers (MR, LMP). All interviews were conducted in German.

In order to provide all participants and organisers with adequate time and space to express their thoughts on the M23C, we chose four different qualitative methods: a narrative interview, FGDs, group interviews and expert interviews.

To explore the experiences, perspectives and motivations around the M23C and how and why it was established we chose the method of the narrative interview for the founder of the M23C. By providing a minimum of structure we aimed to stimulate the participant to reconstruct the initiation and continuation of the M23C.

In order to be able to discuss a range of perspectives on the M23C, we decided to have three internally homogeneous FGDs with different groups of participants (i.e. medical students, other health professionals, organising committee) who presumably had had different experiences with the M23C.

A qualitative group interview took place with two members of the founding organising committee members. We chose the method of a group interview because the number of former participants was not sufficient for an FGD.

We integrated the perspectives and experiences of the panel discussion participants by conducting four expert interviews with an expert or a patient to ensure that they could talk openly about all other experts or patients.

All interviews and FGDs were conducted using semi-structured guides. For the narrative interview we developed an interview guide with i) a narrative stimulus, ii) narrative follow-up questions and iii) closing questions. All other guides contained five sections: i) reasons for attending the M23C, ii) experiences with the M23C, iii) what students learn and how they benefit, iv) how students learn and v) final questions to end the interview. The guide for the M23C committee contained an additional section on vi) organising the M23C.

All guides were pilot-tested before conducting the first interview and FGD. We conducted the nine FGDs and interviews face-to-face in seminar rooms at the university or the hospital to ensure a private atmosphere. Apart from the two researchers and participants, no one else was present. All interviews were audio recorded. MR and LMP took field notes after all interviews and focus groups.

### Qualitative data analysis

All nine audio files were transcribed by MR using f5 transkript [34] and analysed using structured content analysis as described by Schreier [35] using MAXQDA 2020 [36]. Schreier provides a thorough guide throughout the process of qualitative content analysis whereas other authors provide little or no guidance [35]. The coding frame was inductively developed, with coding themes derived from the data [37]. Patterns in the data were recognised by initially sorting data more-than-once-occurring sequences of explanations and by searching for extreme or counterintuitive examples. Starting from a rather descriptive analysis, we started to identify, specific and consolidate emerging patterns within the data. In an iterative process, patterns were consolidated, specified and integrated where it made sense [38]. MR and LMP independently coded one FGD transcript, discussed emergent themes and agreed on an initial coding frame. Thereafter, this coding frame was applied to all transcripts by MR. In the analysis, the different qualitative data were coded using the same frame, while at the interpretation level we paid particular attention to the different perspectives of the participants. The research team met regularly during the study to discuss the analysis. After data analysis was finalised, the category system as well as exemplary quotes were translated into English by MR.

### Quantitative sampling and recruitment

We used a convenience sample of M23C participants during seven medical M23C screenings (see Table 1). There was no additional recruitment for the survey beyond the usual promotional efforts of the M23C (i.e., faculty newsletter, M23C social media, posters, flyers). The survey was distributed in the lecture hall, where the M23C evenings took place, before the film started.

**Table 1:** M23C screenings in the summer semester 2017 and winter semester 2017/2018

Cinema date	Topic	Film	Film director	Release date
23/05/2017	Medical checklists	The Checklist Effect	Lauren Anders Brown	2016
07/06/2017	Blindness	Notes on Blindness	James Spinney	2016
20/06/2017	Migration	On Call	Alice Diop	2016
24/10/2017	AIDS	Dallas Buyers Club	Jean-Marc Vallée	2013
14/11/2017	Forensic psychiatry	Picco	Philip Koch	2010
12/12/2017	Professionalism	The Unknown Girl	Jean-Pierre Dardenne, Luc Dardenne	2016
10/01/2018	Multiple sclerosis	Multiple Fates	Jann Kessler	2016

### Quantitative data collection

Informed by the findings of the qualitative research, we developed a multiple-choice survey with 16 items on reasons for attending the M23C and three items on benefits of attending the M23C. Through qualitative content analysis we derived at a coding frame. The subcategories of the category reflecting on the reasons of attending as well as the benefits of attending were then transformed into quantitative survey items. An example of how this transformation was done can be found in Table 2. One question about dating was added post hoc, informed by discussions in the research team.

**Table 2:** Conversion from qualitative quotes into quantitative items

Qualitative Quote	Qualitative Code	Quantitative Survey Item
And then I just said “Well, then you’ll go when you have time and when the film is good” – so if the film appeals to me and the topic comes up again [...], then the discussion will also be interesting afterwards. – B4, other health professional students, focus group	Interest in film	… have an interest in the film.
Just to meet people, to see what’s interesting. – B2, organising committee, focus group	Interest in getting to know other medical students	… want to meet other medical students.
I think that [...] then the part why you really attend begins. Because I could watch the film [...] at home, too. But I go there to listen to the opinion of the experts. In other words, that’s when the really important part comes, which in my opinion is [...] already too short. – B6, other health professional students, focus group	Interest in discussion	… have an interest in the discussion.
There is [...] often the possibility that we [...] often have experts from LMU or TU (Technical University of Munich) there. [...] It is often the case that after the discussion [...] students go back to the doctors and ask questions, and I think this sometimes leads to a doctoral thesis. [...] By talking to someone in person, [...] it is always something different than when you write an email. [...] This sometimes creates opportunities that you wouldn’t necessarily have otherwise. – B2, organising committee, focus group	Establishing contacts, getting to know people	… want to network.

For the benefits questions we used a five-point Likert scale, with 1 meaning “disagree” and 5 “agree”. Socio-demographic characteristics addressed included gender, age, course of studies, university, education level and participation frequency. Zensus direkt [39] was used to construct the survey.

### Quantitative data analysis

We performed a descriptive analysis (i.e. mean value, standard deviation) and constructed bar and Likert plots in R [40].

### Integration

For the integration of the qualitative and quantitative findings we used the software MAXQDA 2020 and applied the “following a thread” protocol [41–44]. First, we sorted the data by creating a unified list of themes and constructing a convergence coding matrix. Second, we analysed the data in a joint display table by agreement, partial agreement or neutral and disagreement [45]. Third, in a completeness assessment we compared the qualitative and quantitative results and shared the integrated results with the research team for feedback and comment.

### Ethical approval

Ethical approval for the study was obtained from the Ethics Committee of the Medical Faculty of LMU Munich (No. 537–16). All potentially eligible participants were informed about the research in oral and written form. Furthermore, participants in the qualitative component of the study received and had to sign an informed consent form stating that all data would be treated anonymously. An exception was the narrative interview with MS, who agreed that his data could be published non-anonymously. Participants in the quantitative component of the study were informed about the survey in oral and written form and signed an informed consent form. No fees were paid for participation in this study although participants in the qualitative component of the study were taken out for an inexpensive dinner.

## Results

### Characteristics of participants in the qualitative study component

In total, 28 persons were interviewed. Table 3 provides an overview of their characteristics.

**Table 3:** Qualitative study component: Overview and characteristics of participants

Study component	Participants (B1-B8)	n	Age range	Gender	Duration
Narrative interview	Physician	*n* = 1	65	male	50 min
Focus group	Medical students (organising committee)	*n* = 6	20–25	4 female, 2 male	72 min
Focus group	Medical students	*n* = 7	22–25	4 female, 3 male	48 min
Focus group	Three medical-technical radiological assistants in education, two students of psychology, one of biology, one of social work, one nurse in training	*n* = 8	20–25	all female	88 min
Group interview	Physicians (former organising committee)	*n* = 2	28–29	all male	66 min
Expert interview	Affected person (patient or relative)	*n* = 1	47–50	female	50 min
Expert interview	Physician	*n* = 1		male	51 min
Expert interview	Affected person (patient or relative)	*n* = 1		trans	52 min
Expert interview	Physician	*n* = 1		male	83 min
In total:		*n* = 28	20–65	17 female, 10 male, 1 trans	560 min

### Characteristics of the participants in the quantitative study component

Four hundred eighty (95.4%) out of 503 completed questionnaires could be used for the analysis, 18 were excluded due to missing values and five due to incorrect age (we only included participants aged 17 to 90 years). 366 (76.3%) respondents were female, 112 (23.3%) male and 2 (0.4%) chose the “other” option. The mean age (including standard deviation) was 23.1 ± 4.9 years. 336 (70.0%) respondents were medical students, 78 (16.2%) other health students or professionals in training and 66 (13.8%) students from non-health related disciplines. Of all medical students 81 (22.5%) were in the pre-clinical years of medical school, 268 (72.5%) in the clinical years, 10 (2.8%) in internship (last year), and 8 (2.2%) were graduated physicians. Of all participants, 348 (72.5%) studied at LMU Munich, 94 (19.6%) at the Technical University of Munich (TUM) and 38 (7.9%) came from other institutions. 177 (36.9%) participated for the first time, 303 (63.1%) participated at least for the second time.

### Reasons for attending M23C

**Fig. 1:**
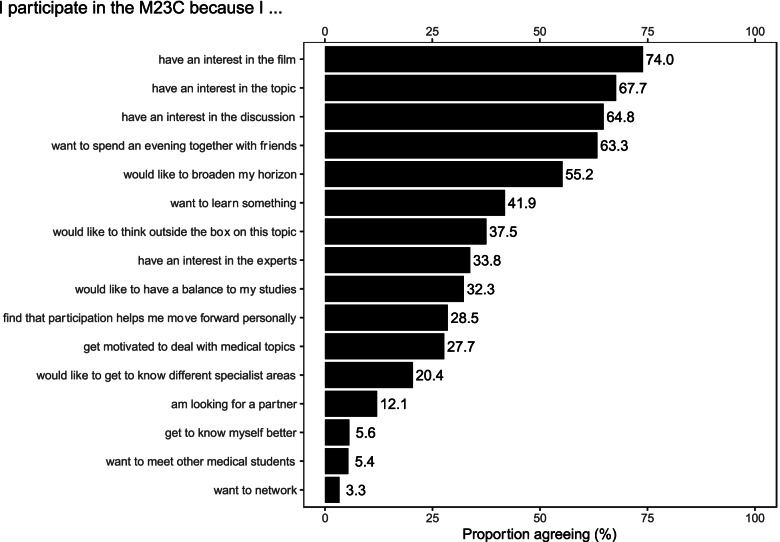
Percentage distribution of reasons for participating in the M23C. Survey responses to multiple-choice question with 16 items of 480 participants in seven M23C courses combined

Reasons for participating in the M23C varied in the qualitative and quantitative component. Figure 1 presents the reasons for attending M23C obtained through the survey, listed in order of importance. More than half of the 480 participants gave one or several of the following five reasons, i.e. that they had an interest in the film (355 or 74.0%), in the topic (325 or 67.7%), in the discussion (311 or 64.8%), that they wanted to spend an evening together with friends (304 or 63.3%) and/or to broaden their horizon (265 or 55.2%). On average, a participant gave 5.7 reasons (SD: 2.96) for attending the M23C. The three reasons that were of least importance to participants were “get to know myself better” (27 or 5.6%), “want to meet other medical students” (26 or 5.4%) and “want to network” (16 or 3.3%).

In general, reasons for attending the M23C were similar among males and females, Interestingly, more male participants reported an interest in spending an evening with friends and to use the M23C as a means of looking for potential partners.

**Fig. 2:**
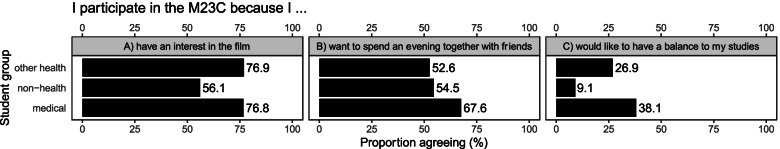
Percentage distribution of reasons by group of students. Survey responses of 480 participants to survey items selected by three subgroups to see differences in these: 336 medical students, 78 other health professional students and 66 non-health professional students

**Fig. 3:**
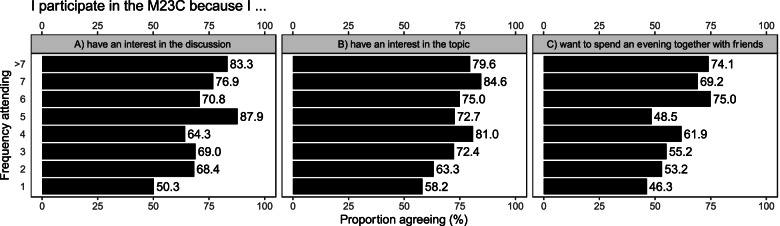
Percentage distribution of reasons compared by frequency of participation. Survey response of 480 participants to one item, by three subgroups to better understand differences in the item for multiple participation: 336 medical students, 78 other health professional students and 66 non-health professional students

In the following, we describe a selection of reported reasons for attending M23C, as well as one surprising reason (i.e. dating as a reason for attending), drawing on survey findings for different sub-groups of participants (Fig. 2 and Fig. 3) as well as insights from the qualitative component of the study.

The joint display table (Table 4) demonstrates the integration of the qualitative and quantitative component. Agreement, partial agreement and disagreement with the item in the qualitative data as well as lack of data for the different items are contrasted with the overall and stratified agreement in the quantitative component.

**Table 4:** Joint display table of integration of qualitative and quantitative components

Item	Qualitative Component			Quantitative Component	
	Agreement	Partial Agreement or Neutral	Disagreement	Overall Agreement	Stratified Agreement
Interest in the film	And maybe these are also films, I think, that I wouldn’t watch in such a private setting, but which I still find totally interesting. And then discussing them with experts afterwards might give me the incentive to say “Cool, I’m actually interested in that. Then I’ll just watch the film. – B5, other health professional students, focus group And then I just said “Well, then you’ll go when you have time and when the film is good – so if the film appeals to me and the topic comes up again [...], then the discussion will also be interesting afterwards. – B4, other health professional students, focus group	not available	Yes, or that you have met a professor or a doctor where you think “Wow, it would be cool to sit there at some point”. [...] because you just know that [...] a cool discussion is going to evolve [...]. So I think that the discussion is sometimes more important than the documentary or the feature film. Then you have to weigh up a bit. – B2, organising committee, focus group, The documentary [is] sometimes not so important. [...] I think the discussion is sometimes more important than the documentary or the feature film. You have to evaluate that a bit. – B2, organising committee, focus group	74.0% agreement	Medical students: 76.8% Other health students: 76.9% Non-health students: 56.1%
Interest in the topic	You sit at home, somehow looking at the topics and thinking to yourself: “such a difficult topic”. So that’s how I feel then. (laughter) That in the evening I don’t really feel like watching a very thoughtful film. And [M23C] is of course a much better set-up. Simply to be able to say that this is M23C, that’s where I’m going now. [...] I know it will be a difficult subject, but I will certainly benefit a lot from participating. I don’t think I would be up for that at home. – B4, organising committee, medical student, focus group; I always thought about a topic that I would find interesting for me personally and looked for it accordingly. – B2, organising committee, focus group … to feel that there is so much interest in the topic. – Affected person, expert interview	I took note [that there is] something like that. And I have planned [...] to see what [...] the next events [are]. For which diseases are the next films? I have to admit, however, [that] it [initially] [...] remained with this intention [...] – Expert, expert interview	I simply decide whether I go [to an M23 cinema evening] at all, based on [whether] it interests me or whether it doesn’t interest me. – B2, health professionals, focus group	67.7% agreement	First-time participants: 58.2% Multiple time participants: 63.3 to 84.6%
Interest in the discussion	I think that [...] then the part why you really attend begins. Because I could watch the film [...] at home, too. But I go there to listen to the opinion of the experts. In other words, that’s when the really important part comes, which in my opinion is [...] already too short. – B6, other health professional students, focus group Well, I think for me, the discussion is always the most beneficial part. [...] But [...] thinking about it, hearing other opinions and, depending on the situation, hearing aspects that you have never even thought about yourself [...] – that is what I personally benefit from the most. – B2, organising committee, focus group	not available	At the very beginning I simply found the film exciting. I didn’t know about the discussion [...] at all. And then I kept on coming back because of the discussions – B6, organising committee, focus group; I was really positively surprised because, like [person] B6, I was not aware of the discussion. And to experience that felt great. – B4, organising committee, focus group	64.8% agreement	First-time participants: 49.7% Multiple time participants: 64.3 to 87.9%
Spend an evening together with friends	I think that with [...] blockbusters, however, a large part of them simply come to see the film with friends and have a nice evening and not primarily because of the discussion. – B6, organising committee, focus group It’s actually always a very cool evening, if you meet up with friends beforehand and then go there [together]. – B5, other health professional students, focus group The first time a friend asked me. And then I thought it was pretty cool. [...] Partly you go because you think “Oh, that film is great. I wanted to see it anyway.” And then why not somehow in a setting like that, where you meet fellow students and can have a discussion about it with experts. That’s actually a pretty good bonus. – B6, medical students, focus group	not available	not available	63.3% agreement	Medical students: 67.6% Other health students: 52.6% Non-health students: 54.5%
Broaden my horizon	I would really see it with a catchphrase [...] as broadening my horizons. Because [it] is rather the possibility to get to know such different perspectives [...] – B4, organising committee, focus group I think it really broadens the horizon somehow. For me at least. Just now [at] “24 weeks” [film]. I didn’t think about how [an abortion] would be carried out. And when you saw it - it’s kind of obvious that the child has to get out somehow. But I wouldn’t have thought that you could take contraceptive drugs and then have to have [the baby] and give birth. But it all makes sense, because it doesn’t disappear just like that. [...] But to see that for once. Not to say “[...] I don’t want the child now, let’s get rid of it, quickly” but to see what was behind it all, I found it quite interesting. – B3, other health professional students, focus group And that not only the medical perspective […] is considered, but also the personal side and what actually happens when the job of a doctor is done. – B6, other health professional students, focus group The fact that there is an attempt being made to bring medicine more in touch with social issues. Or vice versa, to integrate socially relevant topics into medical courses and thus bridge the gap. In other words, to somehow broaden the horizon a little to the left and right. – Expert, expert interview You are not limited to the subject matter alone, as you are in your studies, but [...] you broaden your horizon even further and then receive significantly more input than if it were really just a course per se. – B1, medical student, focus group	not available	not available	55.2% agreement	First-time participants: 46.3% Multiple time participants: 48.5 to 75.0%
Learn something	Whenever I am there, I have learned something afterwards. [...] I learn something from the colleagues. I learn something from the students, from the questions the students ask. Sometimes there are very clever people among them. They really know what they’re doing. You can only kneel down when they make their comments. Or about the patients who are there [...]. Or from the prosthesis maker. You can always learn something. – MS, narrative interview	Yes, as a side effect [...] So I don’t have a bad conscience that I go there now and don’t have to learn. [...] But I can [learn there] or do [it] subconsciously. – B3, organising committee, focus group	not available	41.9% agreement	Medical students: 38.7% Other health students: 52.6% Non-health students: 45.5%
Think outside the box on this topic	And not only the medical side of the whole thing is considered, but also the personal side and what actually happens when the task of the doctors is over. And I’ve often heard from doctors or physicians I’ve been with that they think that the medical side gets far too little attention in their studies. So that’s their opinion, [that] I can’t judge. And I actually think – I hope – that doctors will be able to look beyond this medical edge through the M23 cinema. – B6, health professionals, focus group	not available	not available	37.5% agreement	Medical students: 37.8% Other health students: 41.0% Non-health students: 31.8%
Interest in the experts	You really need [...] a lecturer who has experience in this. So that he or she can also stimulate the discussion a bit. Simply [...] can also guide the discussion a bit towards a few aspects. Where the students don’t just ask questions, but where the lecturer also stimulates a certain discourse. – Expert, expert interview I always find it exciting to see how the different experts perceive the films differently. – Affected person, expert interview [...] I also think it’s really cool [...] that experts come, some of whom are highly respected people, and then really come there [...] and [...] get involved in such a discussion. [...] And that is also simply [...] a [unique] opportunity. – B5, health professionals, focus group	not available	But with the other films, the discussion [...] with the experts didn’t help me that much. – B3, medical students, focus group	33.8% agreement	Medical students: 34.8% Other health students: 34.6% Non-health students: 27.3%
Balance to my studies	I think it’s a great way to balance out the rest of my studies. – B5, organising committee, focus group; I think because it was simply something different. It was in addition to the normal university programme and you could somehow do something nice with your fellow students in the evening. – B1, medical students, focus group	not available	not available	32.3% agreement	Medical students: 38.1% Other health students: 26.9% Non-health students: 9.1%
Helps me move forward personally	The benefit curve [has now] flattened out a bit, but at that time it already helped me enormously, because I [...] was simply in regular exchange with fellow students, with professors about topics, about trisomy 21. – B1, former organising committee, group interview But at that time I think it did help me, definitely. – B1, former organising committee, group interview	not available	not available	28.5% agreement	Medical students: 30.4% Other health students: 26.9% Non-health students: 21.2%
Get motivated to deal with medical topics	You should somehow realise that learning can also be fun. It should not only be [...] about learning, but also about having fun. – B1, former organising committee, group interview I also think [it’s] simply [about] motivation. I was involved in medicine and it was fun. (Laughter) Which is not always necessarily the case. – B6, organising committee, focus group	not available	not available	27.7% agreement	Medical students: 29.5% Other health students: 28.2% Non-health students: 18.2%
Get to know different specialist areas	But then, of course, there are also topics [...] that one has already dealt with a lot [in one’s studies], but perhaps has not [...] had the opportunity to get to know those affected or other disciplines. – B5, organising committee, focus group	not available	not available	20.4% agreement	Medical students: 19.0% Other health students: 23.1% Non-health students: 24.2%
Looking for a partner	not available	not available	not available	12.1% agreement	Female participants: 9.8% Male participants: 19.6% Participants of other gender: 0.0%
Get to know myself better	I don’t know if it really benefits [...] my profession in particular. But I would say that it definitely benefits me, [...] that perhaps one becomes more open [towards] some attitudes [...]. And maybe take other opinions to heart [...]. I think it does bring something, but more for me than for my profession. – B5, health professionals, focus groups	not available	not available	5.6% agreement	Medical students: 7.1% Other health students: 2.6% Non-health students: 1.5%
Meet other medical students	Just to meet people, to see what’s interesting. – B2, organising committee, focus group	not available	not available	5.4% agreement	Medical students: 5.7% Other health students: 5.1% Non-health students: 4.5%
Network	There is [...] often the possibility that we [...] have experts from LMU (Ludwig Maximilian’s University of Munich) or TU (Technical University of Munich) there. [...] It is often the case that after the discussion [...] students go back to the doctors and ask questions, and I think this sometimes leads to a doctoral thesis. [...] By talking to someone in person, [...] it is always something different than when you write an email. [...] This sometimes creates opportunities that you wouldn’t necessarily have otherwise. – B2, organising committee, focus group	not available	not available	3.3% agreement	Medical students: 3.6% Other health students: 1.3% Non-health students: 4.5%

#### Film as a reason

The quality of the film seemed to be of importance, as described by this student:

And then I just said "Well, then you'll go when you have time and when the film is good – so if the film appeals to me and the topic comes up again [...], then the discussion will also be interesting afterwards. – B4, other health professional students, focus group

Students also expect to see films they would otherwise not watch by themselves, which this student elaborates on:

And maybe these are also films, I think, that I wouldn't watch in such a private setting, but which I still find totally interesting. And then discussing them with experts afterwards might give me the incentive to say "Cool, I'm actually interested in that. Then I'll just watch the film. – B5, other health professional students, focus group

Interestingly, one of the organising committee members disagrees with the film itself always being a major reason:

Yes, or that you have met a professor or a doctor where you think "Wow, it would be cool to sit there at some point". [...] because you just know that [...] a cool discussion is going to evolve [...]. So I think that the discussion is sometimes more important than the documentary or the feature film. Then you have to weigh up a bit. – B2, organising committee, focus group

As shown in Fig. 2, 76.8% medical students and 76.9% other health students “have an interest in the film”, compared to 56.1% non-health students. Some participants in the qualitative study elaborated on this in more detail.

#### Topic of the evening as a reason

The M23C seems to offer a safe space for difficult topics:

You sit at home, somehow looking at the topics and thinking to yourself: "such a difficult topic". So that's how I feel then. (laughter) That in the evening I don't really feel like watching a very thoughtful film. And [M23C] is of course a much better set-up. Simply to be able to say that this is M23C, that's where I'm going now. [...] I know it will be a difficult subject, but I will certainly benefit a lot from participating. I don't think I would be up for that at home. – B4, organising committee, medical student, focus group

One of the members of the organising committee argued that the choice of topic was her first priority for organising an event:

I always thought about a topic that I would find interesting for me personally and looked for it accordingly. – B2, organising committee, focus group

One of the invited affected persons who participated in the discussion supported this previously addressed high interest in the respective topic addressed at the respective evening:

… to feel that there is so much interest in the topic. – Affected person, expert interview

Of the first-time participants, 58.2% (103 out of 177) attended the M23C due to an “interest in the topic”, compared to a range of 63.3 to 84.6% of participants who had attended the M23C before (cf. Fig. 3).

#### Discussion as a reason

Some of the first-time participants did not seem to know about the discussion and considered it as a reason to attend several times:

At the very beginning I simply found the film exciting. I didn't know about the discussion [...] at all. And then I kept on coming back because of the discussions – B6, organising committee, focus group

I was really positively surprised because, like [person] B6, I was not aware of the discussion. And to experience that felt great. – B4, organising committee, focus group

This student is very clear that the discussion is her main motivator to attend the event:

I think that [...] then the part why you really attend begins. Because I could watch the film [...] at home, too. But I go there to listen to the opinion of the experts. In other words, that's when the really important part comes, which in my opinion is [...] already too short. – B6, other health professional students, focus group

For another participant the discussion is not only the main reason, but the student also draws a clear benefit from it:

Well, I think for me, the discussion is always the most beneficial part. [...] But [...] thinking about it, hearing other opinions and, depending on the situation, hearing aspects that you have never even thought about yourself [...] – that is what I personally benefit from the most. – B2, organising committee, focus group

Of the first-time participants 49.7% (88 out of 177) attended the M23C due to an “interest in the discussion”, and the percentage was even higher in those participants who repeatedly attended the M23C (cf. Fig. 3).

#### Evening with friends as a reason

There seems to be a group of participants who mainly want to enjoy an evening with friends and see the M23C as an event:

I think that with [...] blockbusters, however, a large part of them simply come to see the film with friends and have a nice evening and not primarily because of the discussion. – B6, organising committee, focus group

Participants repeatedly described that attending the event together with friends contributed to their positive experience:

It's actually always a very cool evening, if you meet up with friends beforehand and then go there [together]. – B5, other health professional students, focus group

The first time a friend asked me. And then I thought it was pretty cool. [...] Partly you go because you think "Oh, that film is great. I wanted to see it anyway." And then why not somehow in a setting like that, where you meet fellow students and can have a discussion about it with experts. That's actually a pretty good bonus. – B6, medical students, focus group

Of the medical students 67.6% (227 out of 336), compared to 52.6% (41 out of 78) of other health professional students and 54.5% (36 out of 66) of non-health professional students attend the M23 because they “want to spend an evening together with friends” (cf. Fig. 2).

#### Broadening one’s own horizon as a reason

The M23C seems to stimulate students to take on other perspectives:

I would really see it with a catchphrase [...] as broadening my horizons. Because [it] is rather the possibility to get to know such different perspectives [...] – B4, organising committee, focus group

I think it really broadens the horizon somehow. For me at least. Just now [at] "24 weeks" [film]. I didn't think about how [an abortion] would be carried out. And when you saw it – it's kind of obvious that the child has to get out somehow. But I wouldn't have thought that you could take contraceptive drugs and then have to have [the baby] and give birth. But it all makes sense, because it doesn't disappear just like that. [...] But to see that for once. Not to say "[...] I don't want the child now, let's get rid of it, quickly" but to see what was behind it all, I found it quite interesting. – B3, other health professional students, focus group

According to some participants, the M23C represents a missing link to the medical humanities:

And that not only the medical perspective […] is considered, but also the personal side and what actually happens when the job of a doctor is done. – B6, other health professional students, focus group

The fact that there is an attempt being made to bring medicine more in touch with social issues. Or vice versa, to integrate socially relevant topics into medical courses and thus bridge the gap. In other words, to somehow broaden the horizon a little to the left and right. – B1, expert interview

This student is very clear that at M23C, she does not only get to know biomedical facts, but gets a variety of information that help her understand the topic from different angles:

You are not limited to the subject matter alone, as you are in your studies, but [...] you broaden your horizon even further and then receive significantly more input than if it were really just a course per se. – B1, medical student, focus group

Of the first-time participants 46.3% (82 out of 177) attended the M23C because they “would like to broaden [their] horizon”. For multiple time participants it ranges from 48.5 to 75.0% (cf. Fig. 3).

#### Balance to curricular studies as a reason

Both the organising committee and the participants see the M23C as a balance to their studies:

I think it's a great way to balance out the rest of my studies. – B5, organising committee, focus group

I think because it was simply something different. It was in addition to the normal university programme and you could somehow do something nice with your fellow students in the evening. – B1, medical students, focus group

Of the medical students 38.1% (128 out of 336), compared to 26.9% (21 out of 78) of other health professional students and 9.1% (6 out of 66) of non-health professional students attended the M23C due to its inherently different character when compared to other subjects in the curriculum (cf. Fig. 2).

#### Dating as a reason

None of the participants in the qualitative study mentioned dating as a reason to attend the M23C.

Of the female participants, 9.8% (36 out of 366) are looking for a partner at the M23C, compared to 19.6% (22 out of 112) of male participants and 0.0% (0 out of 2) of participants of other gender.

### Perceived benefits from participating in M23C

**Fig. 4:**
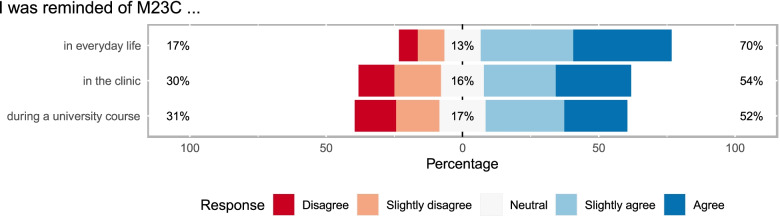
Likert plot of remembering an M23C evening again. Survey response of 480 participants to three items on remembering the M23C evening again with a five-point Likert scale, with 1 meaning “disagree” and 5 “agree”

In the following, we describe insights from the qualitative component of the study as well as the most commonly reported benefits for attending M23C (Fig. 4). Participants reported several benefits from participating in the M23C during other courses in their curriculum, clinical internships or in everyday life. In addition uncertain benefits were reported.

#### Perceived benefits for university course

A medical student remembers exam situations in which they were confronted with content from the M23C:

I actually must say that it has happened to me a few times during exams, that I read something there and something actually rang in the back of my head. And where I thought I had heard that before [in M23C]. – B3, medical student, focus group

More than half of the participants who attended the M23C several times remembered an M23C evening during another university course (cf. Fig. 4, mean: 3.29, median: 4, variance: 1.9, SD: 1.38, on a Likert scale from 1 meaning "disagree" to 5 "agree").

#### Perceived benefits for clinical internships

Participating in the M23C might help a student to remember an illness when they see a patient during a rotation:

Once in the clinical internships [...], where a patient with an illness showed up, which was very rare. Then the doctor said "you probably don't know that" and [I] stood up and said "yes, actually I have heard that before [in M23C]. This has happened a few times now. – B3, medical student, focus group

One medical student shared that he assumes that patients of the soon-to-be doctors might benefit from the broader view, the focus on psychosocial aspects and interdisciplinary approach fostered by M23C:

Also for later [you] remember some situations. If you have patients with dementia, you should also involve their relatives and ask them how you can help them or make suggestions who they can turn to. That you also know of all of these other professions, [...] which you [...] do not know right now – that you can still communicate this to the patient and integrate him into self-care groups and so on [...] – B5, organising committee, medical student, focus group

Participants who attended the M23C several times remembered an M23C evening in the setting of clinical courses, bedside teachings, clinical internships or in their practical year (cf. Figure 4, mean: 3.38, median: 4, variance: 1.93, SD: 1.39, on a Likert scale from 1 to 5).

#### Perceived benefit in everyday life

A former medical student reported that he was able to tell interesting stories by participating in personal conversations at parties:

I remember that half a year later people were talking about this film and then at some party [...] explaining to someone what the film was about, what the expert had said about it, and that this always led to good discussions. – B2, former organising committee, group interview

Participants who attended the M23C several times remembered an M23C evening during everyday life (cf. Fig. 4, mean: 3.83, median: 4, variance: 1. 48, SD: 1.22, on a Likert scale from 1 to 5).

#### Uncertain benefit of attending the M23C

Two participants see a rather limited benefit for their professional life, but at the same time describe an openness towards other attitudes and opinions:

So I don't know whether it really does anything for my profession. But I would say that it definitely does something for me, as I just said, that maybe you become more open in some attitudes towards [...] And maybe you take other opinions to heart or something. I think that does something, but more for me than for my job now. – B5, other health professional students, focus group

The M23C could help them to remember this in the long term. The concrete application of the insights gained seems to be difficult for some participants:

I think it generally just widens the horizon. So I don't know now how I should use it later. Probably in the sense of treating people who had a certain disease and got over it or who are not well with a certain disease. Which was perhaps treated there once. And then you think back to the discussion. But I don't know now how I should implement it concretely. – B6, other health professional students, focus group

## Discussion

### Main findings

To the best of our knowledge, our study represents the first mixed-method study of a cinemeducation course in Germany. It integrates in-depth qualitative insights on why students participate in the M23C and how they benefit from it and largely representative quantitative insights on the extent to which these reasons and benefits play a role for participants. In terms of reasons for attending, we found that the majority of participants attends the M23C due to an interest in the film, the topic and the discussion as well as to spend an evening with peers or to broaden their horizon. Some reasons vary depending on the educational background (medical vs. other health vs. non-health students), participation frequency and, to a lesser extent, gender. In addition, we were able to show that the perceived importance of the discussion increases when the M23C is attended multiple times. With repeated participation, the desire to broaden one’s horizons seems to increase in importance. With respect to likely benefits, we found that the M23C helps to become aware of different perspectives of a disease and to remember a cinema evening or an illness later on, for example, in the clinic during an internship or as a doctor.

At the initiation of the study we presumed that the unique method of the cinemeducation course M23C is the combination of a film with an audience discussion. We hypothesised that a large share of the participants attended because of the possibility to watch a film for free. We were surprised to see that this motivation shifted in participants who had participated several times and who perceived the discussion and the topic as integral. This finding confirms previous research that reported that films can initiate significant group discussions [46, 47].

The M23C pursues an open discussion where the audience – the students – play a critical role in raising questions. This is different from most other cinemeducative approaches: Baños et al. describe that their discussion focuses on questions prepared by the teacher [48]. The open approach of the M23C, that the moderator sparks a discussion among the participants, that the students themselves pose questions that interest them and that the invited guests mainly speak on questions addressed directly to them and do not prepare a presentation, seems to be an essential success factor positively perceived by the participants in this study.

Due to the fact that the search for experts in the organising committee takes up a large part of the time, we assumed that the invited experts were an important reason for participation – similar to the film, discussion and topic – and were surprised that this was rarely explicitly mentioned. However, participants described that the different invited guests had different perspectives on the topic and that they had benefited from the diversity of perspectives in forming their own opinions.

Medical students, despite increasing emphasis on the biopsychosocial model described by Engel in 1977, still seem to notice a lack of “human touch” in their curricular studies. This gap can be filled by voluntary cinemeducation courses like the M23C [49]. Our study showed that the majority of participants use the M23C to widen their horizon which is consistent with other studies [50]. We assume that this broadening of their horizon will enable medical students to recognise different human perspectives on health and illness.

Compared to non-health and other students, medical students seem to attend the M23C more as a balance to their curricular studies and in order to spend an evening together with friends. This could indicate that medical students use the M23C as compensation, but still want to learn something. It could also suggest that medical students have a greater need for compensation to their studies, or that there are currently not enough compensatory events with a psychosocial background in medical studies compared to other studies and health professional trainings. The results, particularly the qualitative components, may imply that it remains difficult for medical students to perceive the need for psychosocial aspects of health and illness as a priority learning experience and that there should be a shift from purely biomedical content to biopsychosocial approaches in medical school courses to foster a holistic overview of health topics. The students’ quotes seem to imply that they undervalue psychosocial elements in their curricula but that when they are exposed to it, e.g. in M23C, they find that they value it highly.

Students reported benefits from participating in the M23C. In general, they linked knowledge acquired in M23C with memories of the film and the discussion, which is consistent with Shankar’s [1] experience. Participation in the M23C could help prospective physicians to consider psychosocial aspects such as support groups and relatives for their patients. In addition, the medium of film with a subsequent peer discussion seems to help participants to remember content from the M23C later on in both professional and private contexts.

To date, it has not been researched how many cinemeducation courses are offered globally. Judging from the literature, a handful of medical cinemas exist in Germany, some of them located at medical faculties and clinics or medical societies with varying target groups. In addition, there are isolated film festivals worldwide with a focus on global health, public health, psychiatry and stigma – often organised outside of the university context. In university courses, whole films or excerpts of films are increasingly used for teaching purposes, although many curriculum designers do not seem to be aware of the research field of cinemeducation [17, 19, 29, 51]. The proportion of non-health students and the open approach, suggest that the cinemeducation methodology as used in M23C can be transferred to other disciplines.

### Strengths and limitations

Due to the fact that we chose a mixed-methods study design and involved most of the organisers of the M23C (idea provider, former and current organising committee) as well as different groups of participants, experts and affected persons, we were able to obtain a comprehensive overview of the M23C. The multiple data sources enabled us to answer our questions in a more nuanced way. For example, we were able to show not only various reasons for participating in the M23C, but also the percentages in which these are important in different groups of participants. Initially, it was planned to conduct one further focus group with medical students. We, however, decided to refrain from further data collection after one focus group with medical students due to data saturation. The design of FGD might have prevented some students from sharing further reasons from participating due to peer pressure; however, we are confident that the risk of social desirability was relatively low. Due to our exploratory sequential design, where the qualitative study component preceded the quantitative study component, we were not aware of the large presence of non-health professional students and did not arrange for further data collection with this subgroup of participants. This lack of representation of non-medical and non-health views in our qualitative data may have led to some reasons for participation or some benefits not being adequately reflected in our survey tool. In light of the qualitative results we might have included “interest in psychosocial aspects of medicine” as a reason in the survey which should be taken in consideration for future research. Integrating a clustering of the qualitative codes in our study design could have resulted in a more in-depth analysis.

By generating the questionnaire and the reasons from the qualitative results and a literature search, we believe that we were able to integrate the most important reasons for participation. In addition, we distributed the questionnaire on seven cinema evenings and can thus ensure that any systematic differences between evenings would not have biased the results.

The data collection was conducted by two researchers (MR, LMP). The participants of some qualitative components may have refrained from mentioning negative aspects of M23C because some of them were acquainted with MR. In order to minimise this risk, we deliberately pointed out at the beginning of the interviews and FGD that positive and negative contents about M23C are of interest and encouraged participants to openly share their perceptions. However, we cannot rule out potential effects of two different interviewers/moderators on the perspectives shared by the participants. Due to the anonymous nature of the survey, it is moreover likely that the 503 surveys were not filled out by 503 different individuals but that some of the participants attending more than one event filled in the questionnaire multiple times.

In order to present our findings to a broad international audience, we had to translate exemplary citations into English. This might have resulted in some loss of meaning. Our study provided rich qualitative and quantitative data. Integration represents a strength of our study in that we were able to use the qualitative quotes to better frame, understand and interpret the quantitative results.

## Conclusions

This study provides an overview of the M23C at the LMU Munich, exploring the different reasons for participants to attend and how it benefits them in their future careers in health care. The M23C is rooted in the combination of film and the subsequent peer and expert discussion that encourages participants to reflect upon their opinions and experiences. Participants seem to value this combination and to benefit by gaining a better understanding of the biopsychosocial aspects of health and illnesses. Furthermore, the film and the discussion also seem to help the participants to remember the contents of the cinema evening at later points in their studies, in a clinical setting or in everyday life.

The study also provides useful insights for planning future cinemeducation courses. It suggests that cinemeducation organisers should above all attach great importance to the selection of films and topics and less to the invited experts. Cinemeducation courses like the M23C could contribute to teaching health professionals a more humane and empathetic way of medicine – something which is still rarely taught in medical school.

## Supplementary Information

**Additional file 1: Supplementary file 1.** Coding frame.

**Additional file 2: Supplementary file 2.** Quantitative dataset of the survey.

## Data Availability

The qualitative datasets used will not be publicly available due to concerns regarding the identification of the participants and in agreement with the ethical approval obtained by the institutional review board based at the medical faculty of LMU Munich. However, these data are secured and could be made available if the request is in line with the data usage outlined in the ethical approval. The quantitative datasets supporting the conclusions of this article are included in the article and its supplementary file. More detailed datasets are available from the corresponding author on reasonable request.

## Abbreviations

M23C: Munich Medical M23 Cinema
LMU Munich: Ludwig-Maximilians-Universität München
MeCuM: Medical Curriculum Munich
FGD: Focus Group Discussion
