# Supplementary material for: Cinemeducation in medicine: a mixed methods study on students’ motivations and benefits
Source: BMC Med Educ. 2022 Mar 12;22:172. doi: 10.1186/s12909-022-03240-x (PMC8918310; doi:10.1186/s12909-022-03240-x)
Supplement: Supplementary file 1 — Additional file 1: Supplementary file 1. Coding frame. [file 12909_2022_3240_MOESM1_ESM.pdf]

**Additional File 1: Coding frame.**

| Coding Frame | |
| --- | --- |
| **Gründe** | **Reasons** |
| Spaß | Fun |
| Interesse am Thema | Interest in the topic |
| Interesse am Film | Interest in the film |
| Interesse an Diskussion | Interest in the discussion |
| Interesse am Konzept | Interest in the concept |
| Interesse Medizinstudierende kennen zu lernen | Interest in meeting other medical students |
| Ausgleich zum Studium | Balance to studies |
| Kontakte knüpfen, Leute kennenlernen | Networking, meeting people |
| Fachgebiete kennenlernen | Getting to know specialties |
| Ergänzung des Pflichtcurriculums | Addition to the compulsory curriculum |
| sich in der Freizeit mit Medizin beschäftigen | Engage in medicine in their free time |
| gemeinsamer Abend mit Freunden | Evening together with friends |
| gemeinsamer Abend mit Gleichgesinnten, Kommilitonen | Evening together with likeminded and fellow students |
| regelmäßige Teilnahme | regular attendance |
| wer einmal da war, fand es gut | who has been there once, liked it |
| Filme würde man nicht in normalem Kino anschauen | Films you would not see in a normal cinema |
| spontan, wenn man nichts anderes vor hat | spontaneous if you do not have anything else to do |

| Coding Frame | |
| --- | --- |
| **langfristige Erinnerung** | **long-term memory** |
| es hört nie auf | it will never stop |
| Thema beschäftigt einen langfristig | Topic occupies you for the long term |
| Gespräch mit Freunden, Kollegen | Conversation with friends, colleagues |
| Situation während Studiums | Situation during studies |
| Situation während Famulatur, Praktisches Jahr | Situation during internship, elective |

Legend: Coding frame used for the qualitative analysis of the reasons and benefits for attending the M23C.
